# Computational proteome-wide screening reveals potential targets of astragaloside IV in *Gallus gallus*

**DOI:** 10.3389/fvets.2026.1887073

**Published:** 2026-07-03

**Authors:** Xiaoqiong Wu, Geng Dong

**Affiliations:** 1 Cancer Hospital of Shantou University Medical College, Shantou, China; 2 Shantou University Medical College, Shantou, China; 3 Department of Pathology, The First Affiliated Hospital of Shantou University Medical College, Shantou, China

**Keywords:** astragaloside IV, MD simulation, molecular docking, molecule interaction, target identification

## Abstract

Astragaloside IV (AS-IV), a primary active constituent of *Astragalus membranaceus*, possesses diverse pharmacological properties, including anti-inflammatory, antioxidant activities. However, its direct molecular targets in poultry have not been fully elucidated. In this study, a proteome-wide target identification strategy was established for *Gallus gallus*, integrating AlphaFold protein structures, P2Rank-based binding pocket detection, and high-throughput reverse docking with UniDock. Following systematic screening, 15 high-confidence candidate targets were prioritized. These targets are associated with lipid metabolism, cell cycle regulation, immune modulation and oxidative stress defense, etc. These findings bridge the gap between empirical observations of AS-IV efficacy and mechanistic understanding at the molecular level. Consequently, this study will accelerate the evidence-based integration of AS-IV into poultry health management strategies.

## 1 Introduction

**Figure 1:**
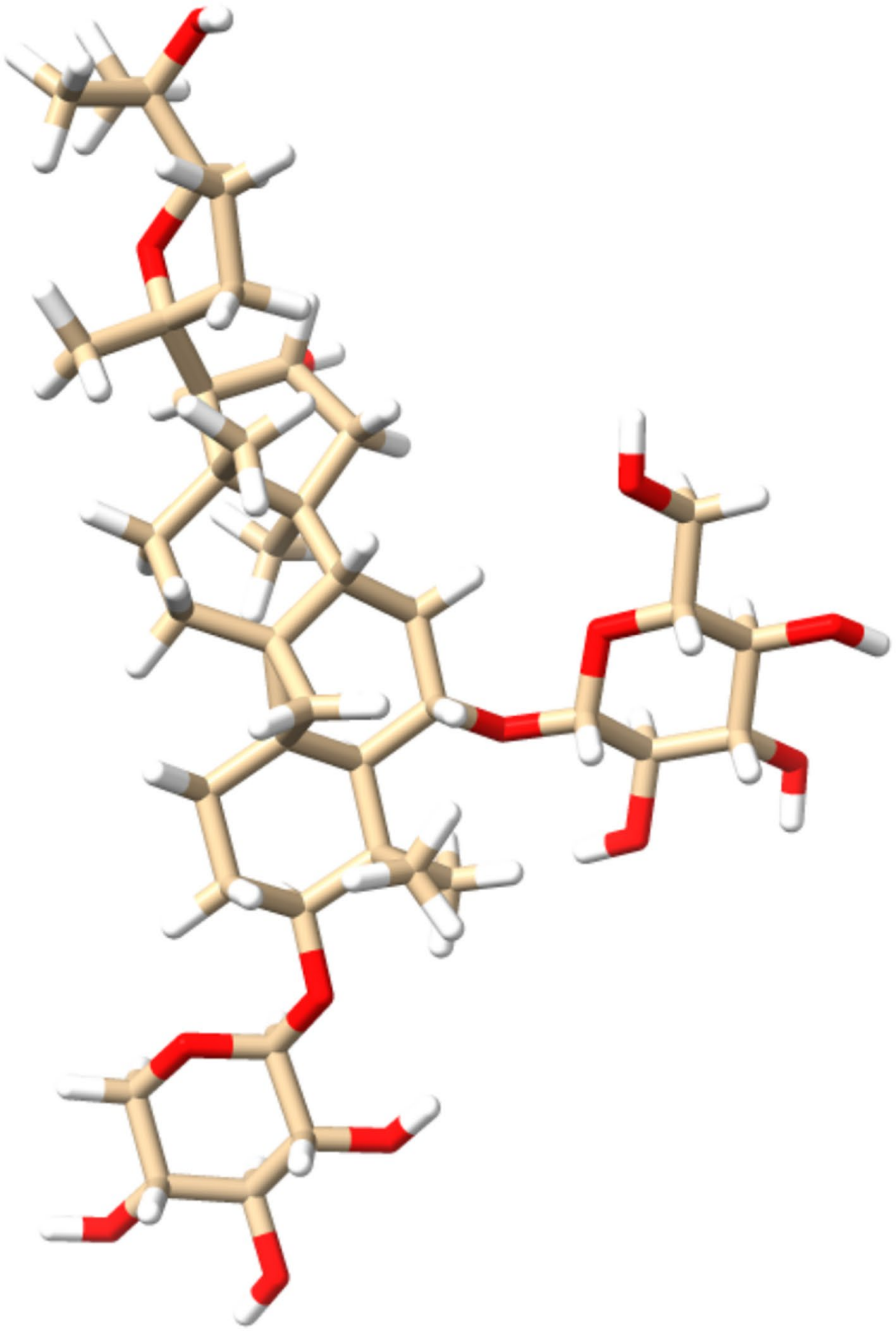
The structure of astragaloside IV (from PDB database (71)).

Natural products derived from *Astragalus membranaceus* have been widely investigated in both human and veterinary medicine because of their immunomodulatory, anti-inflammatory, antioxidant, antiviral, and tissue-protective properties (1–3). Among its major bioactive constituents, astragaloside IV (AS-IV, Figure 1), a cycloartane-type triterpenoid saponin, is considered one of the principal compounds responsible for the biological functions of *Astragalus* preparations (4–6). Accumulating evidence has shown that AS-IV regulates inflammatory signaling, reduce oxidative stress, enhances immune responses, and protects tissues from pathological injury in various disease models (7–12). Because of these broad biological effects, AS-IV has attracted increasing interest as a candidate functional compound for animal health management.

In poultry production, restrictions on antibiotic use are increasing. Therefore, there is growing interest in natural alternatives to conventional antimicrobials and growth-promoting agents for sustainable disease control (13, 14). Phytogenic compounds are particularly appealing because they may enhance host resilience, improve immune competence, and reduce disease-associated losses without directly replacing conventional anti-infective therapy (15–17). *Astragalus*-derived products, including polysaccharides, saponins, and total extracts, have been reported to improve growth performance, antioxidant capacity, and immune responses in *Gallus gallus* (18, 19). In particular, *Astragalus* supplementation has been associated with enhanced antibody production, improved vaccine responsiveness, and modulation of inflammatory cytokine profiles in poultry (20–23).

Previous studies have suggested that *Astragalus*-derived components exert beneficial effects in *Gallus gallus* under conditions of immune challenge, oxidative stress, intestinal injury, and infectious disease (24–28). *Astragalus* preparations have been investigated as supportive agents in vaccination programs and as adjunctive interventions for improving host defense against viral and bacterial challenges (2, 20, 29–31). These observations indicate that AS-IV and related *Astragalus* constituents may have practical value in poultry health promotion and disease management, especially in contexts involving inflammation, epithelial damage, immune imbalance, and stress-associated physiological dysfunction.

Despite these encouraging findings, the direct molecular targets of AS-IV in *Gallus gallus* remain poorly characterized. Most previous studies have focused on phenotypic outcomes, such as immune enhancement, antioxidant activity, or improved disease resistance, while the underlying target proteins and signaling entry points have not been systematically defined. This knowledge gap limits mechanistic interpretation and makes it difficult to rationally position AS-IV in poultry disease intervention. Since natural products often exhibit pleiotropic effects through multiple targets rather than through a single receptor (32, 33), systematic target identification is particularly important. This is especially true for triterpenoid saponins such as AS-IV, whose relatively large and structurally complex scaffold may enable interactions with diverse classes of proteins (34).

Recent progress in artificial intelligence-based protein structure prediction has made proteome-scale target discovery increasingly feasible, even in non-model livestock species. The AlphaFold protein structure database has dramatically expanded structural coverage for protein sequences, including the *Gallus gallus* proteome, thereby enabling large-scale structure-based analysis of compound–protein interactions in *Gallus gallus* (35, 36). In parallel, reverse docking, also known as target fishing, has become a powerful computational strategy to identify potential targets of bioactive compounds by screening one ligand against a large collection of proteins (37–39). This approach has been successfully applied in drug repurposing, off-target prediction, and natural product mechanism research, particularly for compounds with multi-target pharmacology (40, 41).

For poultry disease intervention, identifying AS-IV targets is particularly meaningful because host responses to infection and stress are strongly influenced by inflammatory regulation, mucosal integrity, endocrine signaling, and metabolic homeostasis. AS-IV acts through receptors or regulatory proteins linked to these biological processes. Such targets could explain its reported beneficial effects in *Gallus gallus*. They may also support its use as an adjunctive intervention in disease prevention and recovery. Therefore, in the present study, we established a proteome-wide target fishing workflow to identify potential protein targets of AS-IV in *Gallus gallus*. By integrating AlphaFold-predicted structures (35), P2Rank-based binding pocket prediction (42), and large-scale docking with Uni-Dock (43), we generated a ranked list of candidate *Gallus gallus* targets for AS-IV. These targets were subsequently annotated and interpreted in the context of poultry health and disease modulation.

## 2 Methods

**Figure 2:**
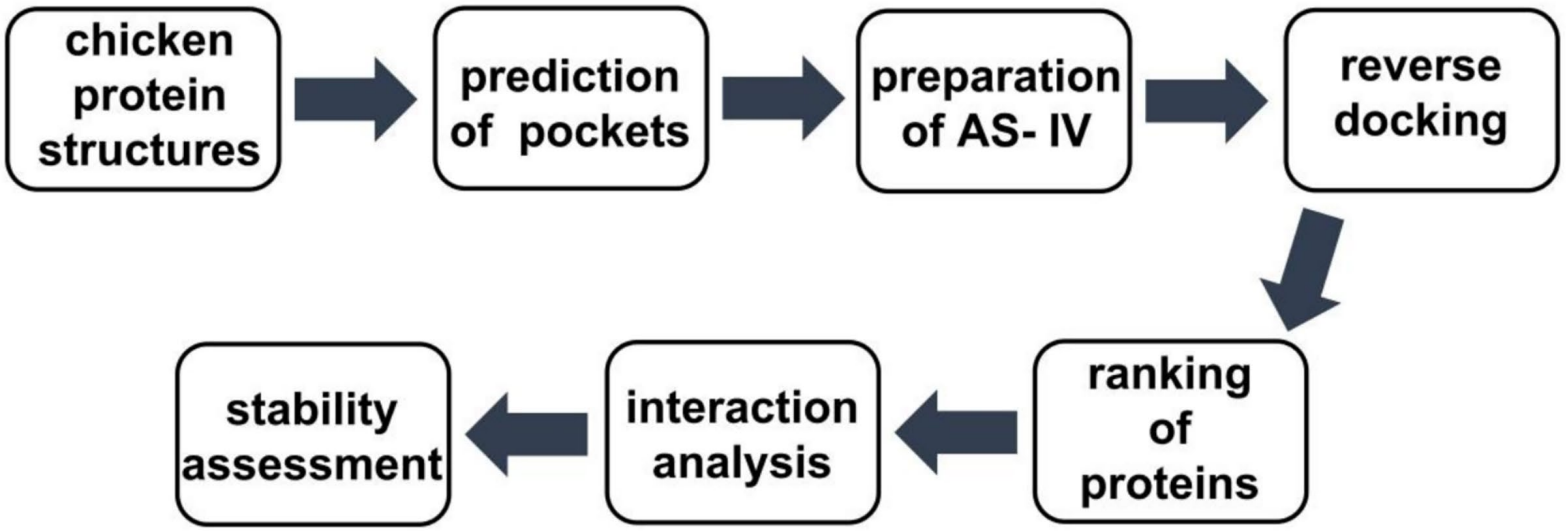
Target fishing workflow in this work.

A proteome-wide target fishing workflow was established to identify potential protein targets of AS-IV in *Gallus gallus* (Figure 2). The workflow consisted of the following steps: (i) collection of predicted *Gallus gallus* protein structures from the AlphaFold protein structure database (35), (ii) prediction of ligand-binding pockets using P2Rank (42), (iii) preparation of astragaloside IV as the query ligand, (iv) reverse docking against the *Gallus gallus* proteome using Uni-Dock (43), (v) ranking and prioritization of candidate targets, (vi) protein–ligand interaction analysis, and (vii) molecular dynamics (MD) simulation for stability assessment of selected top-ranked complexes.

### 2.1 Protein structures

Predicted protein structures of *Gallus gallus* were collected from the AlphaFold Protein Structure Database (35, 36). For annotation, the corresponding protein FASTA dataset was retrieved from UniProt using the *Gallus gallus* taxonomy identifier (Taxonomy ID: 9031). The complete set comprised 46,907 entries. Docking hits were mapped to UniProt entries through accession identifiers and matched FASTA headers, enabling extraction of protein names, gene symbols, review status, and functional annotations.

### 2.2 Binding pocket prediction

Potential ligand-binding pockets for each protein structure were predicted by using P2Rank with default parameters (42). For each receptor, the top-ranked (highest P2Rank score) pocket was selected, and its center coordinates were used for docking box definition. Proteins without valid predicted pockets were excluded from the docking step.

### 2.3 Ligand preparation

The three-dimensional structure of AS-IV was obtained in SDF format and converted into docking-compatible format by generating 3D coordinates, adding hydrogens, and converting to PDBQT format. Open Babel was used for ligand preparation and format conversion (44).

### 2.4 Reverse docking and ranking of candidate proteins

Proteome-wide reverse docking was performed using Uni-Dock (43). For each protein, the docking box was centered on the top-ranked P2Rank pocket. Box dimensions of 30*30*30 Å^3^ were used to accommodate AS-IV. The exhaustiveness parameter was set to 8 (Uni-Dock default). Up to nine binding poses were sampled per protein; only the top-scored pose was retained for ranking. The predicted binding affinity for each successful protein-ligand pair was extracted from the docking output.

Candidate proteins were ranked according to predicted binding affinity, with lower (more negative) docking scores interpreted as stronger predicted binding. Successful docking entries were retained for ranking, whereas proteins without valid pockets or without valid docking output were excluded. The top 50 ranked proteins were selected for downstream interaction profiling.

### 2.5 Protein-ligand interaction analysis

For the top-50 targets, protein–ligand interaction analysis was conducted using the Protein–Ligand Interaction Profiler (PLIP) (45). Docking-pose structures were submitted to PLIP to identify major non-covalent contacts. The analysis focused on hydrogen bonding, hydrophobic interactions, salt bridges, *π*-related interactions, and water bridges between AS-IV and the surrounding residues. Complexes were advanced to MD simulation if they met at least one of the following criteria: ≥10 hydrogen bonds and ≥5 hydrophobic contacts, or ≥15 total non-covalent interactions. This threshold was defined *a priori* to select complexes with chemically plausible, well-supported binding patterns.

### 2.6 Molecular dynamics (MD) simulation

Representative targets were selected from the ranked list based on both docking score and biological plausibility. Priority was given to proteins with known or inferred roles in receptor signaling, transcriptional regulation, intracellular signaling, or disease-relevant regulatory processes. Reviewed entries and proteins with well-defined biological functions were considered particularly informative. Top-50 targets based docking score were used for subsequent MD-based stability analysis and interaction profiling.

To further validate the stability of the docking-derived binding poses, MD simulations were performed for selected representative protein-ligand complexes using the Desmond (Schrödinger Release 2021-1: Desmond Molecular Dynamics System, D. E. Shaw Research, New York, NY, 2021) (46). Each system was solvated in an explicit TIP3P water box. Sodium and chloride counter-ions were added to neutralize the system and achieve a physiological ionic strength of 0.15 M. Each system was energy-minimized (maximum 2,000 steps), then equilibrated using the default Desmond relaxation protocol (NVT and NPT stages). Production simulations of 100 ns were performed in the NPT ensemble at 300 K and 1 atm. All MD simulations were performed using standard procedures identical to that in our previous study (47). The protein-ligand complex was assessed by monitoring the standard root mean square deviation (RMSD) of the protein backbone (Cα atoms).

## 3 Results

### 3.1 Reverse docking and ranking of proteins

A proteome-wide reverse docking workflow was applied to identify potential targets of AS-IV in the *Gallus gallus* proteome. After pocket prediction, docking, and post-processing, a ranked list of candidate targets was obtained based on predicted binding affinity. Following removal of unsuccessful entries, the top-50 ranked proteins were colected in Table 1 with the binding affinities from −24.3 to −13.0 kcal/mol.

**Table 1:** Top-ranked 50 candidate targets of astragaloside IV identified by proteome-wide reverse docking in *Gallus gallus*.

Ranking	Uniprot ID	Gene symbol	Protein name	Binding affinity (kcal/mol)
1	A0A3Q3A4X3	PTPDC1	Protein tyrosine phosphatase domain-containing protein 1	−24.26
2	A0A8V1ALV8	ABR	Active breakpoint cluster region-related protein	−18.68
3	A0A8V0XZB6	FAM120B	Constitutive coactivator of peroxisome proliferator-activated receptor gamma	−18.18
4	P22448	RARB	Retinoic acid receptor beta	−17.41
5	A0A8V0Y9X4	DMGDH	Dimethylglycine dehydrogenase, mitochondrial	−16.63
6	A0A8V1A3V7	HAO2	(S)-2-hydroxy-acid oxidase	−15.37
7	A0A8V0X9R5	IFT80	Intraflagellar transport protein 80 homolog	−15.16
8	A0A8V0ZHU2	NHLRC2	NHL repeat-containing protein 2	−15.04
9	A0A8V0XZ47	SELENOI	Ethanolaminephosphotransferase 1	−14.98
10	A0A8V0X4Q2	SEC14L2	SEC14 like lipid binding 2	−14.86
11	A0A8V0ZT25	TEAD1	TEA domain transcription factor 1	−14.82
12	A0A8V0Z1V0	–	Glycine amidinotransferase	−14.78
13	A0A8V0XPN4	OSBPL1A	Oxysterol-binding protein	−14.61
14	A0A3Q2UG39	OSBPL2	Oxysterol-binding protein	−14.56
15	A0A8V0ZMD2	TLL2	Metalloendopeptidase	−14.53
16	A0A8V0Y3G4	LOC415852	Sulfotransferase	−14.52
17	A0A8V0YM79	FMO3	Dimethylaniline monooxygenase [N-oxide-forming] 3	−14.45
18	A0A8V0Z7M8	NAALADL2	N-acetylated alpha-linked acidic dipeptidase like 2	−14.44
19	E3VVM5	ELOVL1	Elongation of very long chain fatty acids protein 1	−14.33
20	A0A8V0YAJ5	BOP1	Ribosome biogenesis protein BOP1	−14.31
21	P30997	CTNNA2	Catenin alpha-2	−14.26
22	A0A8V0YBJ5	KLHL29	Kelch like family member 29	−13.98
23	A0A8V0XLW6	OSBPL1A	Oxysterol-binding protein	−13.98
24	A0A8V0ZXY2	OSBPL2	Oxysterol-binding protein	−13.89
25	A0A8V0XHR6	KLHL35	Kelch like family member 35	−13.87
26	A0A1L1RM96	PLRG1	Pleiotropic regulator 1	−13.82
27	A0A8V1A4E4	ANAPC5	Anaphase-promoting complex subunit 5	−13.80
28	A0A8V0X768	WSB2	WD repeat and SOCS box containing 2	−13.74
29	A0A8V0XIM9	UGT2A1	glucuronosyltransferase	−13.69
30	A0A8V1A1I5	PGAP3	Post-GPI attachment to proteins factor 3	−13.67
31	A0A8V0YAJ0	BOP1	Ribosome biogenesis protein BOP1	−13.63
32	Q98934	RORB	Nuclear orphan receptor ROR-beta	−13.53
33	Q5ZJW8	DTL	Denticleless protein homolog	−13.40
34	A0A8V0X1T8	BCHE	Carboxylic ester hydrolase	−13.38
35	P79987	HIRA	Protein HIRA	−13.37
36	A0A8V0XAF8	WDR35	WD repeat-containing protein 35	−13.35
37	A0A8V1A2R8	AGPS	Alkylglycerone-phosphate synthase	−13.33
38	A0A8V0XNP3	STRN	Striatin	−13.27
39	Q98TT7	-	Integrin alpha 3A subunit cytoplasmic domain variant	−13.19
40	A0A8V0XXF8	PLA2G7	Platelet-activating factor acetylhydrolase	−13.16
41	A0A8V0Y2B3	KLHL2	Kelch like family member 2	−13.15
42	A0A8V0X7Q7	BCHE	Carboxylic ester hydrolase	−13.15
43	A0A1D5PKL9	ITGA4	Integrin subunit alpha 4	−13.11
44	A0A8V0X6W1	WDR73	WD repeat domain 73	−13.08
45	A0A8V0YFV5	ITGA2	Integrin alpha-2	−13.08
46	A0A8I3AZV5	CASR	Extracellular calcium-sensing receptor	−13.08
47	A0A8V0Z999	LOC100859020	Leucine-zipper-like transcriptional regulator 1	−13.06
48	A0A8V0X4U9	BCHE	Carboxylic ester hydrolase	−13.03
49	A0A8V0Z2J6	ITGA9	Integrin alpha-9	−13.03
50	A0A8V0Y2Y7	KLHL29	Kelch like family member 29	−13.02

An important feature of the ranked target set was the annotation status of the identified proteins. For the top 50 proteins, only four protein were classified as reviewed entries, viz. retinoic acid receptor beta, catenin alpha-2, denticleless protein homolog and protein HIRA. In the UniProt Knowledgebase, reviewed proteins correspond to Swiss-Prot entries, which have undergone manual curation and are generally supported by stronger experimental or literature-based evidence. In contrast, unreviewed proteins belong to TrEMBL, where annotations are primarily generated through automated computational pipelines and homology-based inference (48). Therefore, reviewed entries usually provide higher confidence for direct biological interpretation, whereas unreviewed entries require more cautious evaluation.

**Figure 3:**
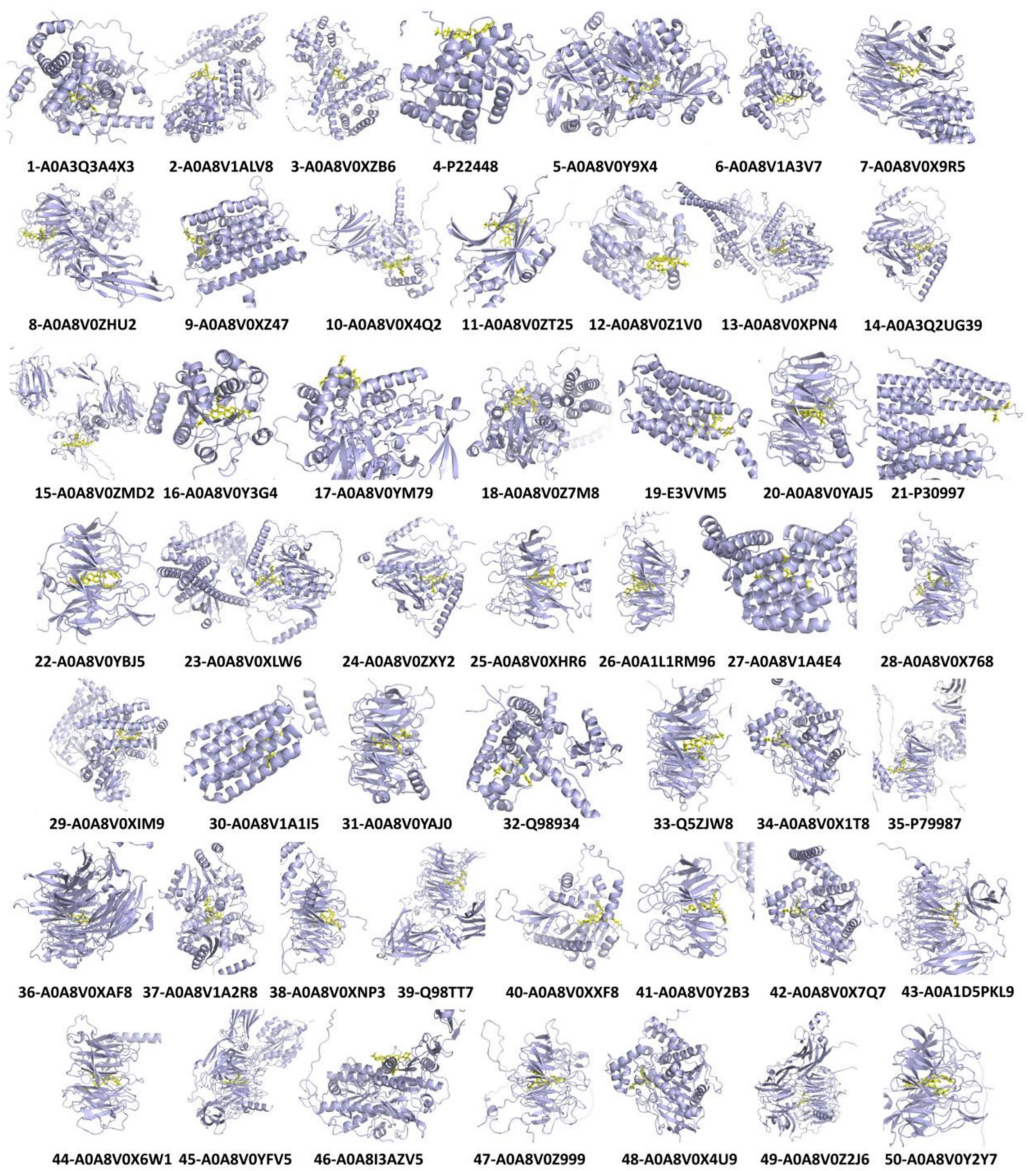
Docking structures of the top 50 ranked candidate protein targets of AS-IV in *Gallus gallus*. Proteins are displayed in cartoon representation, and AS-IV is shown as sticks with yellow.

As is shown in Figure 3, the docking results of the top 50 protein-ligand complexes indicate that AS-IV could be accommodated by a structurally diverse set of *Gallus gallus* proteins. It can be seen that AS-IV was positioned within recognizable cavities, grooves, or recessed regions of the protein structures, except for 12-A0A8V0Z1V0 (glycine amidinotransferase), 15-A0A8V0ZMD2 (metalloendopeptidase) and 46-A0A8I3AZV5 (extracellular calcium-sensing receptor). This results support the structural plausibility of the docking results, and suggest that the reverse docking was capable of identifying protein pockets with potential compatibility for AS-IV binding. Next, the further stability evaluations were performed for the 50 proteins.

### 3.2 Protein–ligand interaction analysis

To further assess the structural plausibility of the docking-derived complexes, protein–ligand interaction profiling was performed for the top-50 ranked targets using PLIP. As is shown in Table 2, the analysis quantified three major classes of non-covalent interactions between AS-IV and the corresponding proteins, viz. hydrogen bonds, hydrophobic contacts, and salt bridges. Considerable variation was observed among the top-ranked complexes, indicating that the quality of ligand recognition differed substantially even within the top docking hits.

**Table 2:** PLIP-based interaction summary of representative top-50 ranked AS-IV-protein complexes.

DockingRanking	Uniprot ID	N_H_bonds_	N_Hydrophobic_	N_Salt_bridges_	N_Total_interactions_
1	A0A3Q3A4X3	10	4	0	14
2	A0A8V1ALV8	10	5	0	15
3	A0A8V0XZB6	7	4	1	12
4	P22448	4	1	1	6
5	A0A8V0Y9X4	6	5	1	12
6	A0A8V1A3V7	5	6	0	11
7	A0A8V0X9R5	9	3	0	12
8	A0A8V0ZHU2	10	4	0	14
9	A0A8V0XZ47	4	2	0	6
10	A0A8V0X4Q2	7	2	1	10
11	A0A8V0ZT25	4	1	1	6
12	A0A8V0Z1V0	6	2	1	9
13	A0A8V0XPN4	5	10	0	15
14	A0A3Q2UG39	3	11	0	14
15	A0A8V0ZMD2	7	3	1	11
16	A0A8V0Y3G4	10	11	0	21
17	A0A8V0YM79	3	5	0	8
18	A0A8V0Z7M8	11	6	0	17
19	E3VVM5	8	8	2	18
20	A0A8V0YAJ5	9	3	0	12
21	P30997	2	8	1	11
22	A0A8V0YBJ5	10	4	0	14
23	A0A8V0XLW6	5	14	0	19
24	A0A8V0ZXY2	3	12	0	15
25	A0A8V0XHR6	13	3	0	16
26	A0A1L1RM96	11	4	1	16
27	A0A8V1A4E4	11	6	0	17
28	A0A8V0X768	7	1	0	8
29	A0A8V0XIM9	7	6	1	14
30	A0A8V1A1I5	4	8	1	13
31	A0A8V0YAJ0	8	3	0	11
32	Q98934	5	7	0	12
33	Q5ZJW8	10	4	0	14
34	A0A8V0X1T8	10	5	0	15
35	P79987	9	3	2	14
36	A0A8V0XAF8	10	0	1	11
37	A0A8V1A2R8	14	6	1	21
38	A0A8V0XNP3	9	2	0	11
39	Q98TT7	4	10	0	14
40	A0A8V0XXF8	4	7	1	12
41	A0A8V0Y2B3	8	3	0	11
42	A0A8V0X7Q7	12	5	0	17
43	A0A1D5PKL9	9	2	0	11
44	A0A8V0X6W1	6	1	1	8
45	A0A8V0YFV5	6	2	0	8
46	A0A8I3AZV5	10	2	0	12
47	A0A8V0Z999	8	3	0	11
48	A0A8V0X4U9	4	5	0	9
49	A0A8V0Z2J6	6	3	0	9
50	A0A8V0Y2Y7	9	4	0	13

The interaction summary revealed that a subset of complexes displayed notably rich interaction patterns. It can be found that 13 candidates exhibited more than 15 total non-covalent contacts, suggesting that AS-IV was accommodated within relatively favorable binding environments. In these cases, the ligand was typically supported by a combination of multiple hydrogen bonds and extensive hydrophobic contacts, consistent with the chemical characteristics of AS-IV, which contains both a large hydrophobic triterpenoid core and multiple oxygen-containing functional groups capable of polar interactions.

Hydrogen bonding is expected to play an important role in stabilizing its binding orientation. 15 protein–ligand pairs formed more than 10 hydrogen bonds. AS-IV contains multiple polar functional groups, including 7 hydroxyl groups and 13 oxygen atoms in total, which provide substantial capacity for hydrogen-bond formation with residues. However, hydrogen bond count alone was not used as the sole criterion for prioritization, as some complexes with moderate hydrogen-bonding patterns still showed extensive hydrophobic complementarity. Hydrophobic contacts were also abundant in many top-ranked complexes, with 13 targets showing more than 5 such interactions. This observation is consistent with the bulky and relatively hydrophobic scaffold of AS-IV and suggests that pocket enclosure and hydrophobic accommodation may be major determinants of stable binding.

In addition, salt bridges observed in selected complexes may provide supplementary electrostatic stabilization. However, they were much less frequent than hydrogen bonds and hydrophobic interactions.

Based on the PLIP interaction profiles, only complexes with relatively rich and well-supported interaction patterns were advanced to the next stage of stability evaluation. Based on the pre-defined PLIP thresholds (≥10 hydrogen bonds, ≥5 hydrophobic contacts, or ≥15 total interactions), 13 complexes qualified for MD simulation: 2-A0A8V1ALV8 (active breakpoint cluster region-related protein), 13-A0A8V0XPN4 (oxysterol-binding protein), 16-A0A8V0Y3G4 (sulfotransferase), 18-A0A8V0Z7M8 (N-acetylated alpha-linked acidic dipeptidase like 2), 19-E3VVM5 (elongation of very long chain fatty acids protein 1), 23-A0A8V0XLW6 (oxysterol-binding protein), 24-A0A8V0ZXY2 (Oxysterol-binding protein), 25-A0A8V0XHR6 (Kelch like family member 35), 26-A0A1L1RM96 (pleiotropic regulator 1), 27-A0A8V1A4E4 (anaphase-promoting complex subunit 5), 34-A0A8V0X1T8 (carboxylic ester hydrolase), 37-A0A8V1A2R8 (alkylglycerone-phosphate synthase) and 42-A0A8V0X7Q7 (Carboxylic ester hydrolase). In addition, the two reviewed proteins 33-Q5ZJW8 (denticleless protein homolog) and 35-P79987 (HIRA), each with total interaction counts within one of the threshold, were also retained given their well-characterized biological functions. In total, 15 complexes were advanced to MD simulation.

### 3.3 MD simulation and stability evaluation

**Figure 4:**
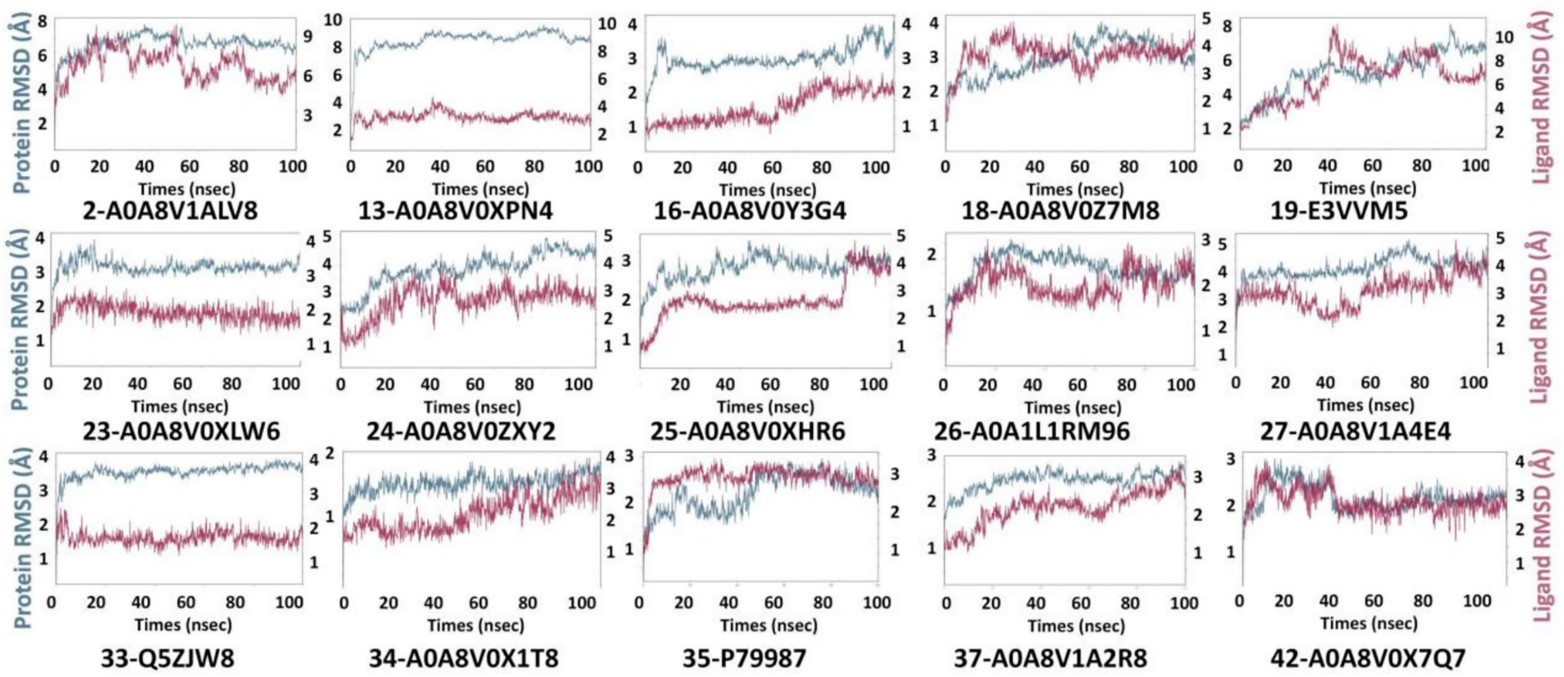
Root mean square deviation (RMSD) of the protein backbone (blue) and astragaloside IV (purple) throughout the 100 ns simulation trajectory for the 15 prioritized candidate targets.

To evaluate the dynamic stability of the 15 prioritized complexes, 100 ns MD simulations were performed. Structural stability was assessed primarily by monitoring the RMSD of the protein backbone and AS-IV heavy atoms (Figure 4). All 15 complexes reached stable equilibrium after an initial relaxation phase. The RMSD values of AS-IV remained relatively low and stable, suggesting that AS-IV was well-retained within the binding pocket without major displacement or dissociation. Specifically, the observed RMSD stability across the 15 prioritized complexes suggests that the docking-derived binding poses represent robust and energetically favorable interaction modes. These results provide computational support for the structural plausibility of the docking-derived binding poses and suggest that the predicted protein-ligand interactions are stable.

## 4 Discussion

The pharmacological effects of AS-IV have been extensively characterized in mammalian and avian models, demonstrating significant potential in anti-inflammation, tissue repair, and immune enhancement (4). However, the molecular targets mediating these pleiotropic effects in *Gallus gallus* have remained largely elusive. In this study, we employed a proteome-wide structure-based target fishing strategy to systematically identify potential binding partners of AS-IV in the *Gallus gallus* proteome. Our results provide a comprehensive target landscape, prioritizing 15 candidate proteins that likely mediate the biological activities of AS-IV in poultry, viz. 1-A0A8V1ALV8 (protein tyrosine phosphatase domain-containing protein 1), 13-A0A8V0XPN4 (oxysterol-binding protein), 16-A0A8V0Y3G4 (sulfotransferase), 18-A0A8V0Z7M8 (N-acetylated alpha-linked acidic dipeptidase like 2), 19-E3VVM5 (elongation of very long chain fatty acids protein 1), 23-A0A8V0XLW6 (oxysterol-binding protein), 24-A0A8V0ZXY2 (oxysterol-binding protein), 25-A0A8V0XHR6 (Kelch like family member 35), 26-A0A1L1RM96 (Pleiotropic regulator 1), 27-A0A8V1A4E4 (anaphase-promoting complex subunit 5), 33-Q5ZJW8 (denticleless protein homolog), 34-A0A8V0X1T8 (carboxylic ester hydrolase), 35-P79987 (HIRA), 37-A0A8V1A2R8 (alkylglycerone-phosphate synthase), and 42-A0A8V0X7Q7 (carboxylic ester hydrolase).

### 4.1 Cell-cycle regulation and genomic stability

HIRA (TUP1-like enhancer of split protein 1, P79987) is a histone chaperone mediating replication-independent deposition of histone H3.3 (49, 50). HIRA plays a critical role in controlling cell growth and regulating cell cycle-related gene transcription (49). In chickens, HIRA has been reported to modulate the expression of histone genes and cell cycle regulators, such as P18, CDC25B, and BCL-2, thereby contributing to the control of cell proliferation and developmental processes (49, 50). Given the regenerative demands of poultry tissue following pathogen-induced injury or inflammatory stress, the ability of AS-IV interact with HIRA suggests a mechanism that AS-IV influence chromatin dynamics and transcriptional programs to support tissue repair. By stabilizing or modulating HIRA-mediated transcriptional networks, AS-IV enhance the cellular capacity to manage proliferative stress and maintain genomic stability in challenged avian tissues.

Denticleless protein homolog (DTL, also known as CDT2; Q5ZJW8) functions as a substrate-specific adapter for the DDB1-CUL4-DTL (also known as CRL4-CDT2) E3 ubiquitin ligase complex (51, 52). This complex is vital for genomic stability, as it mediates the polyubiquitination and subsequent degradation of key proteins like CDT1 and CDKN1A/p21, which are necessary for DNA replication licensing and cell cycle progression (51, 52). Given the constant environmental and infectious stressors faced by commercial poultry, the ability to maintain genomic integrity and proper DNA damage responses is crucial for host resilience. The interaction of AS-IV with CDT2 suggests a potential mechanism for protecting cells against genomic stress. By regulating CDT2-mediated protein turnover, AS-IV helps fine-tune DNA damage responses or circadian clock regulation (53), further supporting the survival and performance of poultry under challenging conditions.

ANAPC5 (A0A8V1A4E4) is a structural component of the APC/C, a multi-subunit E3 ubiquitin ligase. The APC/C orchestrates mitotic exit and G1 maintenance by targeting key cell cycle regulators for proteasomal degradation, including cyclin B, securin, and Polo-like kinase 1 (54). The APC/C has attracted considerable interest as an anti-cancer target because its inactivation leads to mitotic arrest and apoptosis in rapidly dividing cells (55). The identification of ANAPC5 as a candidate AS-IV target is consistent with published evidence that AS-IV induces G2/M cell cycle arrest in multiple cell lines (56).

Pleiotropic regulator 1 (PLRG1; A0A1L1RM96) is a WD40 repeat-containing protein that constitutes a core component of the Prp19 splicing complex (also termed the NTC or complex), which is essential for pre-mRNA splicing fidelity, DNA double-strand break repair, and transcriptional regulation (57). AS-IV may modulate RNA processing and genome stability by interacing with PLRG1.

### 4.2 Lipid metabolism and membrane homeostasis

Three of the candidates belong to the oxysterol-binding protein (OSBP) family (A0A8V0XPN4, A0A8V0XLW6, and A0A8V0ZXY2). OSBPs mediate non-vesicular sterol and phosphatidylinositol-4-phosphate (PI4P) transfer between organelle membranes, regulating cholesterol homeostasis and vesicular trafficking (58, 59). In mammals, dysregulation of OSBP-mediated lipid transfer has been linked to metabolic syndrome, viral replication, and tumor progression (59), making OSBPs an emerging class of druggable targets. Our results show that AS-IV docks favorably into the predicted binding cavities of three paralogous OSBP family members in the *Gallus gallus* proteome, suggesting that modulation of sterol trafficking represents a previously underappreciated mechanism underlying the hypolipidaemic effects of AS-IV. This finding is of particular relevance to poultry production, where excessive hepatic lipid deposition and dyslipidaemia represent major welfare and economic concerns (60).

The candidate E3VVM5, an elongation of very long-chain fatty acids (ELOVL) protein, is equally noteworthy. ELOVL enzymes catalyze the rate-limiting condensation step in the endoplasmic reticulum (ER)-localized fatty acid elongation cycle, thereby governing the composition of very long-chain fatty acids (VLCFAs, ≥C_20_) incorporated into sphingolipids, glycerophospholipids, and wax esters (61). Alterations in ELOVL activity have been associated with neurodegeneration, ichthyosis, and metabolic reprogramming in cancer (62). The potential interaction between AS-IV and an ELOVL enzyme is consistent with previous reports demonstrating that AS-IV attenuates ER stress (63), although direct evidence for ELOVL engagement remains to be established experimentally.

Alkylglycerone-phosphate synthase (AGPS; A0A8V1A2R8) catalyzes a pivotal step in the biosynthesis of ether lipids, including plasmalogens and platelet-activating factor precursors, within peroxisomes. AS-IV is widely recognized as an antioxidant. Yet its potential to engage AGPS and perturb ether lipid biosynthesis has not been explored. This novel hypothesis merits biochemical investigation.

The two carboxylic ester hydrolase candidates (A0A8V0X1T8 and A0A8V0X7Q7) are members of the *α*/*β*-hydrolase superfamily, which encompasses acetylcholinesterase, carboxylesterases, and lipases involved in the hydrolysis of ester bonds in lipids, drugs, and xenobiotics (64). Carboxylesterases play important roles in the metabolism of ester-containing prodrugs (65). AS-IV binding to carboxylesterase may modulate its enzymatic activity, potentially affecting the bioactivation of ester-based therapeutics and altering drug efficacy or toxicity profiles.

### 4.3 Immune modulation and signaling

The active breakpoint cluster region-related protein (ABR; A0A8V1ALV8) is a dual-function RhoGEF/GAP protein. ABR has been implicated in the regulation of cytoskeletal dynamics, neuronal migration, and phagocytosis, and has recently emerged as a modulator of innate immune signaling (66). The predicted binding of AS-IV to ABR raises the possibility that cytoskeletal remodeling and Rho-dependent signaling contribute to the anti-inflammatory and immunomodulatory effects of AS-IV.

### 4.4 Oxidative stress

Kelch-like family member 35 (KLHL35; A0A8V0XHR6) belongs to the KLHL subfamily of BTB-Kelch proteins that function as substrate-specific adaptors for Cullin3 (CUL3)-based E3 ubiquitin ligases (67). KLHL proteins recognize specific degron sequences in target proteins and present them for ubiquitination, thereby controlling the stability of factors involved in oxidative stress responses (e.g., NRF2/KEAP1 axis), muscle differentiation, and autophagy. Interestingly, AS-IV has been shown to activate NRF2-mediated antioxidant signaling in multiple cell types, and competitive displacement of NRF2-related substrates from KLHL/CUL3 complexes represents a plausible molecular mechanism (68–70).

### 4.5 Limitations and future perspectives

Several limitations of this study should be acknowledged. The candidate AS-IV targets were identified through computational inverse molecular docking, which is subject to scoring function limitations and modeling of protein dynamics. Additionally, functional annotations were largely derived from mammalian literature, and species-specific differences may affect their applicability to *Gallus gallus*.

Future validation efforts should prioritize two key objectives: experimental confirmation of biophysical binding, and functional assessment in disease-relevant models. Additionally, pharmacokinetic studies should determine AS-IV tissue distribution and bioavailability, while potential drug-herb interactions with veterinary medicines warrant systematic evaluation. Integration of these complementary approaches will progressively refine the AS-IV target landscape. This will enable evidence-based optimization of AS-IV as a multi-target adjunctive intervention in poultry health management.

## 5 Conclusion

This work presents the first proteome-wide, structure-based virtual screening of AS-IV against the *Gallus gallus* proteome. By integrating AlphaFold-predicted structural models with systematic molecular docking, 15 candidate targets were identified. These include two well-characterized SwissProt-reviewed proteins (P79987, Q5ZJW8) and 13 previously uncharacterized TrEMBL-annotated proteins, spanning lipid metabolism, cell-cycle regulation, immune modulation, and oxidative-stress pathways. The findings offer a molecular-level elucidation for the pleiotropic pharmacology of AS-IV, and could provide the mechanistic basis necessary for the evidence-based optimization of AS-IV application in commercial poultry production. As this study is based on computational predictions, further experimental validation will be essential to confirm the identified targets and elucidate their biological relevance and mechanisms of action.

## Data Availability

Publicly available datasets were analyzed in this study. This data can be found here: https://zenodo.org/records/21123008?token=eyJhbGciOiJIUzUxMiJ9.eyJpZCI6IjYzYTZkNTJiLWFkOTAtNDkyZS1hMTM5LTdlZDQxOWIyN2M0NCIsImRhdGEiOnt9LCJyYW5kb20iOiJjNWYzYzU0OGIxNTViYzE0ZjcxZjk2YTcxMDBhMjNiOSJ9.HF11NOB6byZ54fyHgQHwA22nT1gCg09R8S9PdpOheZ5E63-FNNhAhz9JGH00qRO-RSpJEC3mR5V0yqu8kQD8OQ.
